# Detección de inclusiones citoplasmáticas gigantes en un paciente pediátrico con infecciones recurrentes: a propósito de un caso

**DOI:** 10.1515/almed-2024-0016

**Published:** 2024-02-14

**Authors:** Leire Saiz-Sierra, Anna Marull Arnall, Javier Nieto-Moragas, Meritxell Deulofeu, Orlando Jiménez Romero, Irene Mademont, María Obón Ferrer, María Teresa Serrando Querol

**Affiliations:** Laboratori Territorial ICS-IAS Girona, Hospital Universitari Doctor Josep Trueta, Girona, España; Grupo de investigación en Anatomía Clínica, Embriología y Neurociencia (NEOMA), Departamento de Ciencias Médicas, Universidad de Girona, Girona, Cataluña, España; Comisión de Biología Hematológica de la SEQC, Barcelona, España

**Keywords:** inclusiones gigantes, Chédiak–Higasi, trastorno genético

## Abstract

**Objetivos:**

En algunos casos de infecciones, es frecuente observar inclusiones gigantes en los leucocitos. Sin embargo, estas inclusiones también pueden estar relacionadas con trastornos genéticos raros, como el síndrome de Chédiak–Higashi (SCH). Para realizar un diagnóstico diferencial entre estos grupos de patologías, es necesario realizar estudios genéticos específicos. Dada la dificultad y especifidad de la confirmación diagnóstica, es esencial analizar los antecedentes clínicos y patológicos del paciente, con el fin de obtener una orientación diagnóstica.

**Caso clínico:**

Presentamos el caso de un paciente de tres años de edad con infecciones respiratorias recurrentes. Cabe señalar la presencia de un mechón de cabello blanco en la parte frontal de la cabeza, así como algunas áreas de hipopigmentación en la piel. En el frotis sanguíneo, destacó la presencia de grandes gránulos citoplasmáticos en todos los leucocitos, especialmente en los neutrófilos.

**Conclusiones:**

El SHC es un trastorno genético poco frecuente, causado por una mutación en el gen *LYST* situado en el cromosoma 1q42.3, que codifica la proteína LYST. También se puede realizar un estudio genético molecular para detectar las variantes bialélicas del gen *LYST*. Ante resultados alterados en los valores cuantitativos y/o cualitativos en el hemograma completo, el primer paso del algoritmo diagnóstico incluye realizar un frotis de sangre periférica.

## Introducción

El síndrome de Chédiak–Higashi (SCH) es una inmunodeficiencia autosómica enfermedad recesiva rara descrita por Beguzz (1943), Steinberk (1948), Chédiak (1952) e Higashi (1954) [1–3]. El SCH está relacionado con la presencia de variantes genéticas patogénicas en homocigosis o heterocigosis compuesta en el gen *LYST* (MIM *606897). El gen *LYST* es un gen regulador del tráfico lisosomal [4, 5], localizado en el locus cromosómico 1q42.3, que codifica la proteína LYST [3]. LYST es una proteína adaptadora, implicada en la regulación, fisión y secreción de vesículas intracelulares, como el lisosoma y también puede regular el tráfico de efectores relacionados con la exocitosis [5–7]. Así mismo, la proteína LYST participa en la regulación del tamaño y número de gránulos líticos de las células T citotóxicas y células Natural Killer (NK), también llamadas células asesinas naturales, (De igual forma, esta proteína está implicada en la producción de citocinas proinflamatorias por parte de los macrófagos y las células dendríticas, regulando las vías de señalización endosómicas. LYST también induce una desregulación de la actividad proteína quinasa, ocasionando una disfunción inmunitaria y fenotipos anormales [5–8].

Se han descrito menos de 500 casos de SCH en la literatura [1] y su prevalencia aún no se ha establecido, probablemente debido a la heterogeneidad del trastorno y la ausencia de confirmación genética en muchos de los casos. Así mismo, no se han documentado diferencias entre regiones geográficas o continentes. Se trata de un síndrome que se suele diagnosticar en edad pediátrica, ya que las manifestaciones clínicas se suelen observar en este periodo [9]. El SCH (MIM #214500) está relacionado con las variantes patogénicas del gen *LYST*, y se caracterizan por albinismo oculocutáneo parcial, fotofobia, nistagmo, alteraciones de la coagulación, manifestaciones neurológicas variables y alteración del sistema inmune, especialmente de las células NK, lo que causa una predisposición a sufrir infecciones víricas y bacterianas. Dichos signos se derivan de alteraciones funcionales en las células polinucleares, que contienen grandes inclusiones lisosómicas características de esta patología [9–11]. El SCH tiene una forma de presentación clínica clásica y otra atípica. Los pacientes con la forma clásica tienen mayor probabilidad (hasta el 8,5 % de los pacientes) de desarrollar una fase acelerada, también conocida como linfohistiocitosis hemofagocítica (LHH), que puede resultar fatal, ya que puede provocar fallo orgánico y muerte, si no se trata adecuadamente [5, 9, 12, 13]. Las infecciones recurrentes que se observan en pacientes afectos de CHS se producen por la desregulación proteica de gránulos secretores presentes en neutrófilos. Por otro lado, el albinismo se produce como resultado del déficit de tirosinasa en los melanocitos, debido a la exocitosis y a la presencia de melanosomas gigantes que impiden el transporte de la melanina [3].

El SHC se puede detectar en el frotis sanguíneo o en la médula ósea, por la presencia de gránulos citoplasmáticos gigantes en células como los leucocitos. También se pueden realizar estudios genéticos moleculares para detectar las variantes bialélicas del gen *LYST*.

Las infecciones recurrentes se suelen tratar con antibióticos empíricos, aunque el tratamiento debería establecerse en consonancia con los resultados de los cultivos. Dependiendo de la evolución del paciente, también pueden surgir otras complicaciones. El tratamiento más apropiado para este síndrome es el trasplante de células madre hematopoyéticas o el trasplante de médula ósea. Sin embargo, estos abordajes no tratan los aspectos neurológicos de la enfermedad [13].

Presentamos el caso de un niño de tres años con SCH, cuyo primer hallazgo fue la presencia de gránulos citoplasmáticos gigantes en un análisis de sangre rutinario.

## Caso clínico

Se trata de un niño de tres años de edad que se presentó en su centro de atención primaria por fiebre y síntomas de herpes en los labios, boca y lengua en las últimas 48 h. Sus familiares comentaron que sentía dolor al comer. El paciente tenía antecedentes de infecciones respiratorias recurrentes, que requirieron tratamiento antibiótico, y se encontraba en seguimiento debido a un retraso en el desarrollo psicomotor.

En el examen físico, se observaron múltiples herpes en la cavidad oral, el paladar y la superficie de la lengua. Cabe señalar la presencia de un mechón de cabello blanco en la parte frontal de la cabeza, así como algunas áreas de hipopigmentación en la piel.

Como información relevante sobre su historia clínica, debe tenerse en cuenta la consanguinidad de sus padres biológicos, lo que incrementaba la posibilidad de un amplio espectro de enfermedades hereditarias. Así mismo, el pediatra que realizaba el seguimiento al paciente señaló que el hermano mayor, un niño de cinco años en el momento del caso clínico, presentaba un cuadro de discapacidad intelectual y retraso en el desarrollo psicomotor (dificultad en la psicomotricidad fina, temblores y características dismórficas), que requería atención profesional periódica. En el caso del hermano mayor, se disponía de los resultados de estudios moleculares previos, en los que se detectaron alteraciones cromosómicas: una ganancia de 3 Mb de material genético intersticial en el brazo largo del cromosoma 5, que afecta a la región cromosómica 5q21.3, y una ganancia de 2.9 Mb de material genético intersticial en el brazo largo del cromosoma 9, que afecta a la región cromosómica 9q21.33q22.2. Ambas alteraciones eran *de novo* y se clasificaron como variantes de significado incierto;se desconocen las implicaciones clínicas que han tenido actualmente en el hermano.

**Figura 1:**
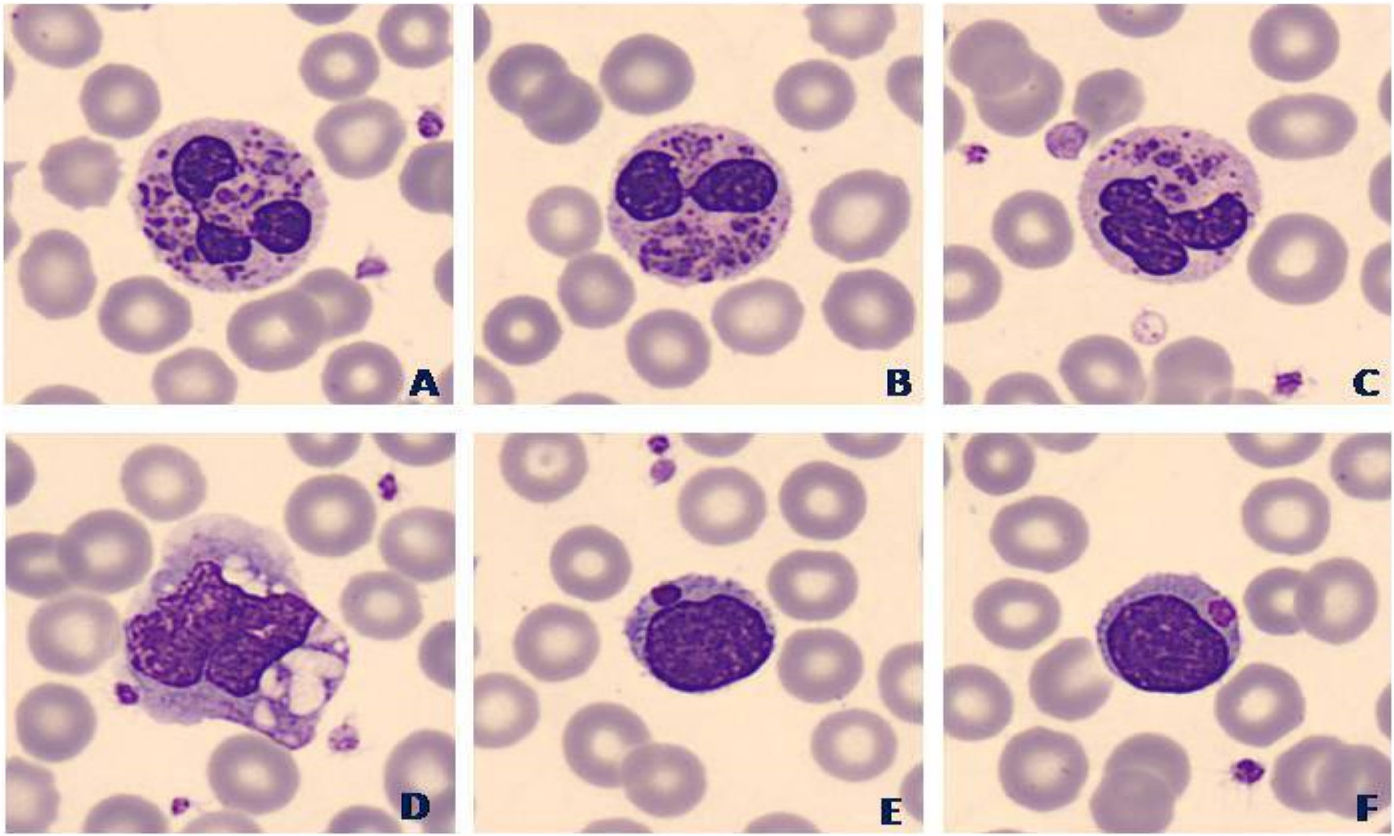
Imágenes de células del frotis de sangre periférica del paciente, en las que se pueden observar inclusiones gigantes en los neutrófilos (A–C), monocitos (D) y linfocitos (E–F).

Los resultados bioquímicosapenas mostraron anomalías en los parámetros analizados. La proteína C reactiva (CRP) estaba alterada, mostrando valores por encima del rango de normalidad (CRP=7.1 mg/dL), además, cabe destacar que la hormona estimulante de la tiroides (TSH) no estaba alterada (TSH=5,03 mU/L). En los resultados hematológicos del del laboratorio, se observó una neutropenia moderada (0.97×10^3^/µL) con monocitosis (1.72×10^3^/µL), que podría estar relacionada con las infecciones recurrentes. Tal como muestra la Figura 1, si observamos el frotis sanguíneo con el microscopio, se pueden observar gránulos grandes o inclusiones citoplasmáticas en la totalidad de los leucocitos, especialmente en los neutrófilos, los linfocitos y los eosinófilos. Estos hallazgos se pueden encontrar en múltiples patologías, incluyendo las infecciones o algunos síndromes hereditarios.

Ante la sospecha de alguna de las anomalías hereditarias relacionadas con la presencia de inclusiones intracitoplasmáticas gigantes, examinamos la historia clínica del paciente y de su familia. En la orientación diagnóstica, así como en la selección de pruebas confirmatorias, buscamos datos del paciente que pudieran ser relevantes para establecer un diagnóstico. Los resultados genéticos y moleculares del hermano mayor suscitaron la sospecha de que las características clínicas del paciente podrían deberse a la presencia de alteraciones cromosómicas. Teniendo en cuenta los hallazgos citológicos y clínicos del paciente, la primera sospecha diagnóstica fue SCH. Así, realizamos un análisis personalizado del exoma centrado en el fenotipo, priorizando los cambios detectados en los genes asociados al fenotipo hematológico observado (*LYST*, *HPS1*, *MLPH*, *MYO5A*, y *RAB27A*), y en los genes relacionados al término Ontología del Fenotipo Humano (HPO): neutropenia (HP:0001875), trastorno del lenguaje (HP:0002463), retraso en el desarrollo del habla y el lenguaje (HP:0000750), problemas en la psicomotricidad fina (HP:0007010), retraso en el desarrollo de la psicomotricidad fina (HP:0010862), temblor (HP:0001337), y retraso en el neurodesarrollo (HP:0012758). El paciente resultó ser homocigótico para la probable variante patogénica NM_000081.3:c.11173G>A p.(Gly3725Arg) en el gen *LYST*. Tanto los padres como el hermano presentaron la variante en heterocigosis, por lo que ninguna presentaba repercusión clínica alguna.

Hasta la última revisión de laevolución clínica del paciente,éste se mantenía con un buen estado de salud con el tratamiento antibiótico profiláctico, y aguardaba un trasplante de células madre de un donante no emparentado.

## Discusión

El síndrome Chédiak–Higashi (SCH) es una enfermedad genética rara de transmisión autosómica recesiva causada por una mutación en el gen *LYST*, localizado en el cromosoma 1q42.3 y que codifica la proteína LYST (gen regulador del tráfico lisosomal) [12]. La probabilidad de transmisión entre familiares varía dependiendo de la relación con el caso índice. Los padres biológicos suelen ser portadores heterocigóticos de una mutación o de variantes patogénicas del gen *LYST*. En la literatura, se han descrito dos pacientes con SCH provocado por una disomía uniparental del cromosoma 1 [14, 15]. Para los descendientes, existe un riesgo del 25 % de no verse afectados por la mutación, un riesgo del 25 % de padecer SCH, y una probabilidad de hasta el 50 % de ser portadores heterocigóticos asintomáticos. Los portadores heterocigóticos permanecen asintomáticos, siendo improbable el desarrollo de la enfermedad. Los descendientes directos del caso índice serán portadores heterocigóticos asintomáticos. De este modo, es necesaria la confirmación genética de los padres y hermanos [3, 9].

El estudio diagnóstico de SCH se debe iniciar tras observar la presencia de gránulos gigantes en el citoplasma de los leucocitos, especialmente de los granulocitos, tanto en la sangre periférica como en la médula ósea [3]. En dichos casos, es preciso realizar un estudio genético y molecular para detectar variantes bialélicas del gen *LYST*. La secuenciación de nueva generación (NGS, por sus siglas en inglés) es una de las estrategias más frecuentemente empleadas para detectar pequeñas deleciones e inserciones intragénicas, mutaciones con cambio de sentido, sin sentido y de empalme del gen *LYST* [3, 4, 10]. En el caso que aquí presentamos, se realizó un análisis mutacional mediante NGS, en el que se analizaron y priorizaron los cambios detectados en los genes asociados a su fenotipo clínico (*LYST*, *HPS1*, *MLPH*, *MYO5A*, and *RAB27A*), así como los posibles cambios patogénicos asociados.

La presencia de inclusiones en el citoplasma de los leucocitos puede estar relacionada con distintas patologías, como infecciones bacterianas (normalmente, en los neutrófilos), infecciones víricas (normalmente localizadas en las células T o las células NK), aunque también se encuentran en síndromes de inmunodeficiencia congénita, concretamente, SCH o el síndrome de Alder–Reilly. En ambos síndromes de inmunodeficiencia, se pueden observar inclusiones gigantes anormales, por lo que será necesario realizar un diagnóstico diferencial. Entre los hallazgos citológicos del SCH, se ha identificado estructuralmente, la presencia de vesículas lisosomales gigantes endiversos componentes celulares (neutrófilos, linfocitos, eosinófilos o monocitos) [7, 8]. En la Tabla 1 se muestran las patologías genéticas más relevantes relacionadas con estos hallazgos microscópicos. Teniendo en cuenta que existen multitud de síndromes raros de etiología genética, así como la elevada heterogeneidad clínica, como discapacidad intelectual, neutropenia o inmunodeficiencia (infecciones recurrentes), es necesario realizar un diagnóstico diferencial.

**Tabla 1:** Diagnóstico diferencial en la aparición de gránulos citoplasmáticos gigantes en las células de sangre periférica.

Patología	Características	Citología	Células afectadas	Gen afectado
Granulación tóxica	Infección o inflamación, embarazo, G-CSF			
Chédiak–Higashi	Inmunodeficiencia, anemia, neutropenia, ictericia, patología neurológica, infecciones recurrentes	Gránulos gigantes con tinción variable	Leucocitos	*LYST*
Alder–Reily	Mucopolisacaridosis	Granulación gruesa similar a la tóxica	Neutrófilos, eosinófilos, linfocitos y, raramente, monocitos	Gen mieloperoxidasa (*MPO*)
Alteraciones relacionadas con MYH9	Trombopenia y plaquetas gigantes	Similar a los cuerpos de Döhle	Neutrófilos, eosinófilos, monocitos, basófilos	*MYH9*
Hermansky–Pudlak	Fibrosis pulmonar; albinismo; diátesis hemorrágica secundaria a disfunción plaquetaria			*HPS1*
Griscelli	Hipomelanosis, hipopigmentación, canas, cabello gris, alteraciones neurológicas e inmunológicas.	Granulación eosinófila gruesa	Granulocitos (neutrófilos y eosinófilos)	*RAB27A*

HPS1, proteína 1 del syndrome de Hermansky-Pudlak ; proteína Rab-27ARAB27A relacionada con Ras.

Así, el panel prediseñado realizado en este caso incluye alteraciones en los genes de síndromes similares, como el síndrome de Alder–Reily, alteraciones en el gen *MYH9*, mucopolisacaridosis y neutropenia [13, 14].

Podemos concluir que, ante la presencia de cambios en los valores cuantitativos y/o cualitativos en el hemograma completo, es esencial realizar un frotis de sangre periférica. El hallazgo de gránulos gigantes en los leucocitos, especialmente en los granulocitos, puede orientar el diagnóstico hacia un grupo de patologías de etiología genética y hereditaria, que es necesario identificar y diagnosticar en el paciente índice y sus familiares. Finalmente, ante la sospecha de este grupo de patologías, está indicada la confirmación diagnóstica mediante análisis citogenético y molecular, con el fin de establecer un diagnóstico confirmatorio y proporcionar asesoramiento genético familiar.
